# Effects of Prebiotic Yeast Mannan on Gut Health and Sleep Quality in Healthy Adults: A Randomized, Double-Blind, Placebo-Controlled Study

**DOI:** 10.3390/nu16010141

**Published:** 2023-12-31

**Authors:** Reiko Tanihiro, Masahiro Yuki, Masaki Sasai, Akane Haseda, Hiroyo Kagami-Katsuyama, Tatsuhiko Hirota, Naoyuki Honma, Jun Nishihira

**Affiliations:** 1 Core Technology Laboratories, Asahi Quality and Innovations, Ltd., Moriya 302-0106, Japan; masahiro.yuki@asahi-qi.co.jp (M.Y.); masaki.sasai@asahi-qi.co.jp (M.S.); tatsuhiko.hirota@asahi-qi.co.jp (T.H.); 2 Department of Medical Management and Informatics, Hokkaido Information University, Ebetsu 069-8585, Japannishihira@do-johodai.ac.jp (J.N.)

**Keywords:** yeast, mannan, prebiotic, gut health, fecal metabolomics, sleep

## Abstract

Human gut health is closely related to sleep. We aimed to evaluate the efficacy of yeast mannan (YM) in improving bowel habits and sleep quality, along with metabolomics in fecal samples. A total of 40 healthy adults (age range, 22–64 years) with discomfort in defecation were enrolled and randomly allocated to receive either YM (*n* = 20; 1.1 g/day) or placebo (*n* = 20) for four weeks. Participants recorded their defecation habits throughout the test periods. Sleep electroencephalogram (EEG) recording using an EEG device and fecal sampling were performed pre- and post-treatment. The YM group significantly increased defecation frequency and stool volumes compared to the placebo group. After 4 weeks of treatment, the non-REM sleep stage 3 (N3) duration in the YM group was significantly higher than that in the placebo group. YM ingestion significantly lengthened total time in bed (TIB) and significantly shortened N3 latency compared to placebo intake during the trial. The metabolomics analysis found a total of 20 metabolite differences between the YM and placebo groups. As a result of stepwise linear regression, changes in fecal propionate and gamma-aminobutyric acid (GABA) levels were identified as the primary factors explaining changes in TIB and N3 latency, respectively. Our findings suggest that the prebiotic YM could be beneficial to gut health and sleep quality.

## 1. Introduction

The human intestinal microbiome is a highly complicated ecosystem, in which several hundred microbial species consume, produce, and exchange hundreds of metabolites [1]. This diverse ecosystem contributes to multiple functions, including maturation of the host immune system, vitamin synthesis [2], degradation of dietary fibers, and maintenance of gut homeostasis [3]. Gastrointestinal microbes produce various bioactive metabolites from food ingredients and endogenous substances, which play an important role in the crosstalk between the host and microbiota [4]. Imbalances in the gut environment encompassing the gut microbiome and metabolites resulting from antibiotics, chronic stress, or a disordered diet can trigger loss of gut function, affecting not only physical but also mental health; therefore, the maintenance of the gut environment is essential for many aspects of health, including gut health [5]. One possible approach to actively modulate the gut environment is through a prebiotic, which is defined as a substrate selectively utilized by host microorganisms to confer health benefits [6]. The clinical application of prebiotics has received increasing attention because of their low risk of serious side effects, ease of administration, and high potential to influence the composition and function of intestinal microbiota. We previously found that yeast mannan (YM) promotes the growth of specific *Bacteroides* in both in vitro and in vivo studies [7,8,9]. YM is a soluble, indigestible carbohydrate originating from the yeast cell walls and consists of highly branched mannose polymers with molecular weights ranging from 20 to 200 kDa [10]. YM is composed of an α-1,6-mannoside backbone and side chains of α-1,2-mannoside and α-1,3-mannoside [10,11]. This composition is different from that of plant-derived mannans, such as carob seed mannan and konjac mannan, which consist of 1,4-β-mannoside bonds [12]. YM is utilized by specific gastrointestinal bacteria due to its highly complex polysaccharide structure [7,8,13]. Thus, the effect of YM on the gut environment has attracted significant research attention. Our previous results demonstrated that YM supplementation can enhance the abundance of *Bacteroides thetaiotaomicron*, a potential novel probiotic, and decrease the abundance of *Ruminococcus*, *Ruminococcaceae;g_*, and *Clostridiales;f_;g_*, without affecting diversity in healthy women [9]. Additionally, YM intake promoted microbial equol production and improved skin dryness [9]. *B. thetaiotaomicron* has attracted attention as a potential probiotic for the relief of Crohn’s disease [14], but the effective use of increasing *B. thetaiotaomicron* has not yet been found in many areas.

An increasing number of people suffer from sleep problems owing to their nocturnal lifestyle and shorter sleep duration [15]. Recently, much evidence has been accumulated regarding the close relationship between the gut and sleep [16,17,18]; for example, it has been reported that the sleep rhythm is altered in mice in which the gut microbiota is removed with antibiotics [19]. In this study, we noticed the impacts of YM on sleep quality, because YM has been found to be effective in altering intestinal microflora in our previous studies [8,9]. Metabolomics, as the technique that captures metabolites which is the most downstream of the central dogma, can bring insight into the more direct effects of the food ingredients on the host’s condition, as compared to the 16S rRNA gene sequencing data that we had obtained in the previous studies [8,9]. Therefore, we assessed the effects of YM administration on gut health and sleep quality in combination with fecal metabolomics to develop further health benefits of YM. To our knowledge, this is the first report of the beneficial effects of prebiotics on objective sleep quality.

## 2. Materials and Methods

### 2.1. Study Design

This was a double-blind, randomized, placebo-controlled, parallel study. Participants were recruited at the Department of Medical Management and Informatics, Hokkaido Information University (Hokkaido, Japan) and randomly allocated 1:1 to each group. The allocation was conducted by a third-party organization using block randomization. The assignment was blinded by participants, researchers, evaluators, and physicians until the investigation was completed. During this study, physical examination, blood sampling, and medical interviews were performed three times: the pretrial test, 0- and 4-week visits. Dietary surveys were conducted at 0- and 4-week visits. Before both 0- and 4-week visits, fecal samples and EEG data were collected. The participants were directed to take five YM/placebo tablets once a day during the intervention. The daily logs included information on bowel movements, tablet intake, menstrual status, physical status, and the use of other medications. The study was conducted from May 2021 to November 2021. The primary outcomes were defined as bowel habits assessed by the daily defecation log, and the secondary outcomes were defined as sleep quality and fecal properties assessed by quantitative PCR and metabolomics based on capillary electrophoresis time-of-flight mass spectrometry (CE-TOFMS).

### 2.2. Participants

The study participants included healthy Japanese adults between 20 and 64 years of age with discomfort in defecation. Sample size was calculated with G*Power 3.1 [20]. Assuming an effect size of 0.85, an α of 0.05, and a β of 0.2 from the previous study [21], the required sample size was calculated to be 36. The number of participants was set to 40, with an expected dropout rate of 10%. Supplementary Table S1 lists the inclusion and exclusion criteria for the participants. Participants were directed to maintain their lifestyle habits and prohibited from taking healthy foods and supplements.

### 2.3. Tablets

YM was prepared from yeast cell walls by Asahi Group Foods, Ltd. (Tokyo, Japan), as previously reported [7]. Five tablets, equivalent to a daily dose, contained 1.1 g of YM, and these were confirmed to contain 0.62 g of mannan using a previously reported measurement method [8]. For the preparation of placebo tablets, the same method was used for active tablets, except that YM was replaced with maltose. Calcium stearate, silicon dioxide, and crystalline cellulose were used to prepare both the YM and placebo tablets.

### 2.4. Bowel Habits and Analyses of Fecal Samples

All participants were directed to record daily defecation frequency, stool volume, and stool form. Stool volume was compared with a ball-shaped model 40 mm in diameter. The stool form was evaluated according to the Bristol Stool Scale (BSS) [22] as type 1 to 7: 1, separate hard lumps, such as nuts; 2, sausage-shaped but lumpy; 3, similar to a sausage but with cracks on the surface; 4, similar to a banana, smooth and soft; 5, soft blobs with clear-cut edges; 6, fluffy pieces with ragged edges, a mushy stool; and 7, watery, no solid pieces.

Fecal samples were collected per participant at the start and end of the intervention. The samples were frozen in the freezers after collection and brought to the study site during the visits. These samples were analyzed for metabolite composition and microbiota composition. Analyses using CE-TOFMS were performed using the Agilent CE-TOFMS system (Agilent Technologies, Waldbronn, Germany) with procedures developed by Soga et al. [23,24,25] at Human Metabolome Technologies (HMT) (Yamagata, Japan). Briefly, approximately 50 mg of fecal sample was dissolved in internal standards (H3304-1002, HMT) and ultrapure water, homogenized using a voltex mixer, and centrifuged. The supernatant was ultrafiltered using a 5-kDa cutoff centrifugal filter (UltrafreeMCPLHCC, HMT); 80 uL of the filtrate was mixed with 20 uL of Milli-Q water. The obtained CE-TOFMS data were processed using MasterHands (Keio University, Yamagata, Japan) to extract peak information, such as migration time (MT), m/z, and peak area. After excluding the peaks of isotopomers, adduct ions, and other product ions derived from known metabolites, the remaining peaks were annotated based on the HMT database. For peak annotation, the tolerance range for MT was ± 0.5 min and that for m/z was ± 10 ppm. The relative levels of each metabolite were calculated using sample amounts and internal standards. Data processing, normalization, and univariate and multivariate analyses of fecal metabolite data were performed using MetaboAnalyst 5.0 [26]. Missing values were replaced with 1/5 of the lowest positive value for each metabolite. The data were mean-centered and divided by the standard deviation (SD) of each metabolite during the normalization process. Principal component analysis (PCA), partial least squares discriminant analysis (PLS-DA), and hierarchical cluster analysis (HCA) were conducted with normalized metabolite data. The variable importance in the projection (VIP) value of each variable in PLS-DA was calculated to represent its contribution to discrimination. Metabolites with a VIP value > 1.0 were further applied to the Student’s *t*-test at the univariate level to determine the significance of each metabolite. Statistical significance was set at *p* < 0.05. Heatmap was generated using the Euclidean distance matrix with the Ward clustering algorithm.

DNA isolation from fecal samples was performed by beads beating previously described [27]. Quantitative PCR (qPCR) analyses were performed as previously described [9,28,29]. Supplementary Table S2 describes the amplification methods and primer sequences. For each absolute quantification, a synthesized DNA fragment with the same sequence as the 16S rRNA gene sequence was used as a reference.

### 2.5. Analyses of Sleep Quality

Sleep EEG was measured by a patch-type EEG device (HARU; PGV Inc., Tokyo, Japan). The reliability of this device has already been confirmed by comparison with the International 10–20 system [30]. The EEG data were collected for three consecutive nights before both 0- and 4-week visits. The following parameters were obtained for each participant: total time in bed (TIB), sleep efficiency (SE), N3 duration, sleep onset latency (SOL), and N3 latency. At the scoring sleep stages, each 30 s epoch of recording epoch was classified into the following five stages according to the AASM2007 manual [31]: wakefulness, REM sleep, and non-REM stages N1, N2, and N3, with comparable accuracy to clinical polysomnography devices [32].

### 2.6. Diet Survey and Safety Evaluation

The food frequency questionnaire (FFQ) was used to assess dietary and nutrient intake. Safety was assessed using diary records, medical interviews, general blood tests, and physical examinations. All adverse events (AEs) and side effects were monitored in participants who took the test tablets at least once, and the incidence of these events was calculated. Blood pressure, pulse rate, and body weight were measured at every visit, whereas body height was measured only at the first visit. Blood samples for general biochemical examinations were obtained in a fasting state at each visit and were measured for the following items at Sapporo Clinical Laboratory Inc. (Sapporo, Japan): white blood cells (WBC), red blood cells (RBC), hemoglobin (Hb), platelet count (Plt), and hematocrit (Ht) for complete blood count test; total cholesterol (TC), high-density lipoprotein cholesterol (HDL-C), low-density lipoprotein cholesterol (LDL-C), and triglyceride (TG) for lipid panel tests; fasting blood glucose (FBG) and hemoglobin A1c (HbA1c) for blood glucose profile test; creatinine (CRE), uric acid (UA), and blood urea nitrogen (BUN) for renal function assessment; and gamma-glutamyl transpeptidase (γ-GTP), alanine aminotransferase (ALT), aspartate aminotransferase (AST), lactate dehydrogenase (LDH), and alkaline phosphatase (ALP) for liver function assessment. A medical interview was conducted by a clinical physician at every visit.

### 2.7. Ethics Committee

The study protocol was approved by the Bioethics Committee of Hokkaido Information University (Hokkaido, Japan) per the principles of the Declaration of Helsinki (approval date: 28 April 2021; approval number: 2021-13). The study was registered with the University Hospital Medical Information Network (UMIN) Clinical Trial Registry as UMIN000044175 (date of registration: 11 May 2021). The study involved one revision of the clinical trial protocol. The contents were (1) the postponement of the study due to the declaration of a state of emergency following the spread of the COVID-19 infection and (2) changes to the case-fixing conditions following the postponement of the study. The changes were approved by the Bioethics Committee of Hokkaido University through an expedited review (approval date: 11 June 2021; approval number: 2021–2024). Written informed consent was obtained from all participants before enrollment.

### 2.8. Statistical Analysis

Statistical analyses were performed using SPSS version 25 software (IBM Japan Ltd., Tokyo, Japan). For intergroup comparison, parametric tests were performed using the Student’s *t*-test and non-parametric tests were performed using the Mann–Whitney U-test. The chi-square test was used to compare the incidence of AEs and side effects between the groups. A multi-linear model analysis with twenty metabolites was performed using the stepwise method. Differences were considered significant at *p*-values < 0.05.

## 3. Results

### 3.1. Demographics

**Figure 1:**
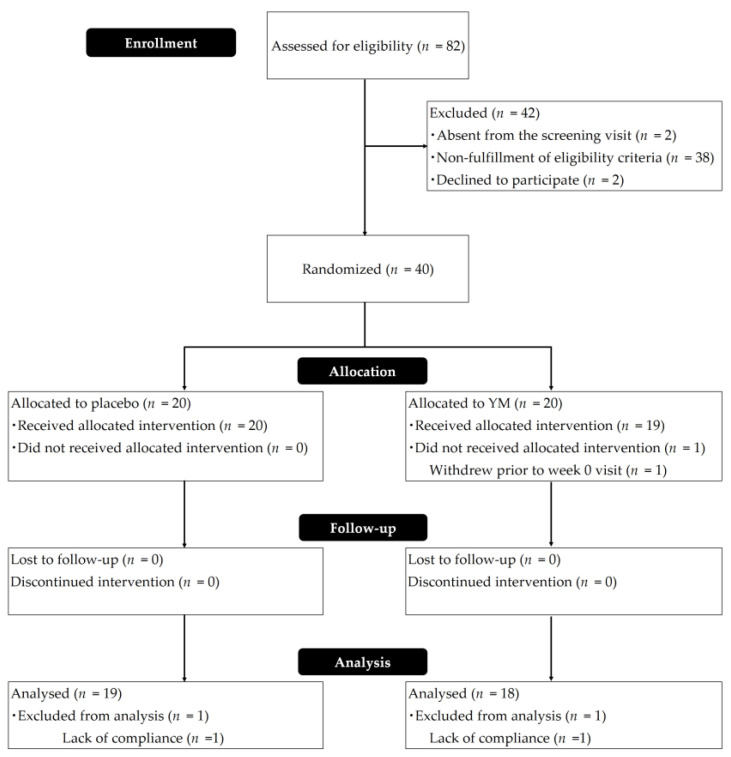
CONSORT flow diagram.

Eighty-two participants were initially recruited, out of which forty-two were excluded and randomly assigned to the YM or placebo treatment. Figure 1 depicts the participant flow diagram. One participant did not receive the assigned intervention for personal reasons. Thirty-nine participants (20 in the placebo group and 19 in the YM group) completed the study and were followed up during the intervention. Two participants (one in the placebo group and another in the YM group) were excluded from the efficacy analysis because they violated the prohibition against eating healthy food. Therefore, data from 37 participants (19 in the placebo group and 18 in the YM group) were used for efficacy analysis. One participant in the placebo group was excluded from sleep analysis because EEG data from week 0 were not available. Thus, efficacy analysis was used in a per-protocol analysis set. The data from the 39 participants who took YM/placebo tablets at least once were used, and safety analyses included incidents of AEs and side effects. Table 1 shows the baseline characteristics of the participants (per protocol). No significant differences between the groups were observed in age, gender, alcohol drinking habits, smoking history, or fiber intake. The frequency (%) of intake of supplementary tablets was 98.41 ± 2.80 and 98.87 ± 0.61 (mean ± standard deviation (SD)) for YM and placebo, respectively, indicating that high compliance was achieved in this study. Dietary surveys using the FFQ showed no changes in dietary or nutrient intake during the intervention period in either group.

### 3.2. Bowel Movements, Stool Volume, and BSS

**Figure 2:**
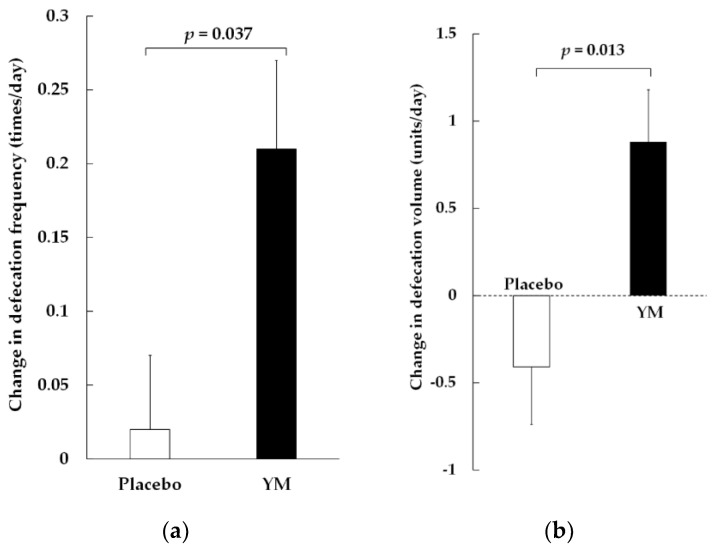
Effects of YM on bowel habits. (**a**) Changes in defecation frequency; (**b**) changes in stool volume. Data are expressed as mean ± standard error (SE). The *p*-values were measured using the Mann–Whitney U-test.

Changes in defecation frequency (times/day) during the treatment period were significantly greater in the YM group than in the placebo group (Figure 2a). The defecation frequency (times/day) at pre-treatment was 0.75 ± 0.06 in the YM group and 0.81 ± 0.05 in the placebo group (mean ± standard error (SE)). Thus, the oral intake of YM helped normalize defecation habits and approach daily bowel movements. Changes in stool volume during the treatment period were significantly greater in the YM group than in the placebo group (Figure 2b). However, the BSS scores of the two groups were not significantly different. (Supplementary Table S3). The distribution of baseline BSS scores in this study was concentrated around the optimal score of 4 (3.5 ± 0.2).

### 3.3. Sleep

The sleep quality was evaluated using an EEG device, and the results were summarized in Table 2. After four weeks of treatment, the N3 duration in the YM group was significantly longer than that in the placebo group, although the baseline N3 duration was not significantly different between the two groups. Likewise, the N3 latency in the YM group was significantly shorter than that in the placebo group after four weeks of intervention, whereas the groups showed no significant difference at baseline. Both the SE and SOL showed no significant differences between the groups. Comparing changes from baseline, YM intake significantly lengthened TIB and significantly shortened N3 latency compared to placebo intake.

### 3.4. Fecal Properties

**Figure 3:**
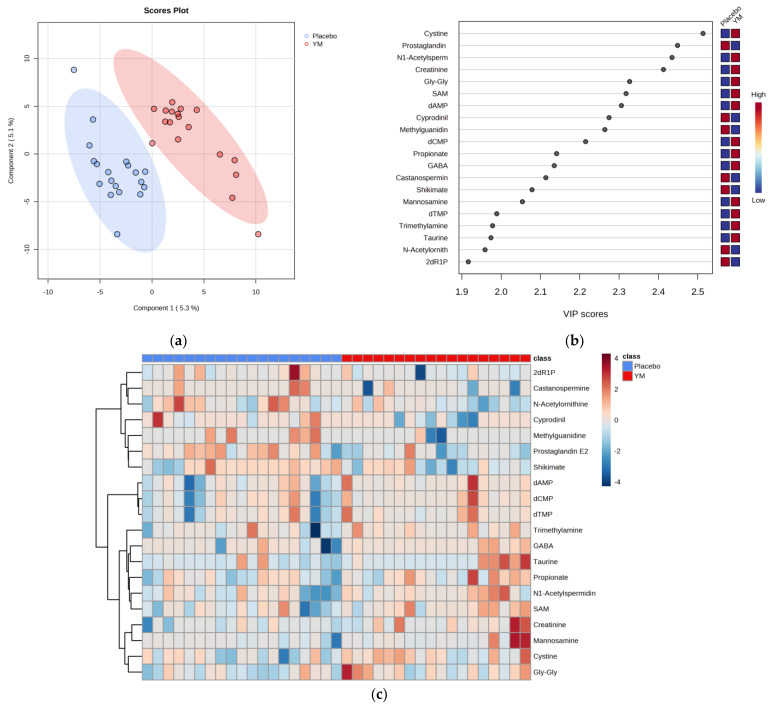
Effects of YM intake on the fecal metabolome. (**a**) PLS−DA plot of the YM (red dots) and placebo groups (blue dots) based on changes from baseline. The ellipses in the score plot illustrate the 95% confidence intervals of the groups. (**b**) The highest VIP values for the first component of the PLS−DA. Red and blue boxes on the right indicate increases and decreases in the metabolite levels, respectively. (**c**) Heatmap of 20 differential metabolites (VIP > 1.0, *p* < 0.05) clustered by YM and placebo groups. The color key represents the Z score.

Changes in both copy number (copies/g feces) and relative abundance (%) of *B. thetaiotaomicron* in the YM group were greater than those in the placebo group. However, no significant changes in total bacterial copy number were observed between the groups (Table 3). CE-TOFMS identified 479 metabolites from fecal samples collected pre- (0w) and post-treatment (4w). Supplementary Figure S1 shows the score plots of principal component analysis (PCA) based on the metabolome dataset from all samples (placebo 0w, placebo 4w, YM 0w, and YM 4w)There was no obvious separation between the four groups based on the PCA analysis. To see differential metabolite changes between the two types of treatment during the trial, partial least squares-discriminant analysis (PLS-DA) was used. Figure 3a shows the score plots of PLS-DA between the groups based on the changes in metabolite levels from pre-treatment (0w). Consequently, plots of the PLS-DA scores demonstrated visible clustering and clear separation between the YM and placebo groups. There were 166 features with VIP values calculated to be greater than 1.0. Figure 3b displays the features with the highest VIP values for the first component of PLS-DA, which are considered important for discriminating between the two groups. To explore metabolites that significantly differed in their changes between the two groups during the trial, we focused on 166 features (VIP > 1.0). The changes in relative level from pre-treatment (0w) were compared between the two groups using the Student’s *t*-test. Differential metabolites were defined using thresholds of VIP >1.0 in PLS-DA and *p* < 0.05 in the Student’s *t*-test. As a result, 20 differential metabolites between groups were found (Supplementary Table S4). Moreover, 13 of these metabolites, namely, cystine, *N*^1^-acetylspermidine, creatinine, glycyl-glycine (Gly-Gly), *S*-adenosylmethionine (SAM), deoxyadenosine monophosphate (dAMP), deoxycytidine monophosphate (dCMP), propionate, gamma-aminobutyric acid (GABA), mannosamine, deoxythymidine monophosphate (dTMP), trimethylamine, and taurine, were significantly increased throughout the intervention in the YM group compared to those in the placebo group. The remaining 7 of 20 metabolites, namely, prostaglandin E_2_, cyprodinil, methylguanidine, castanospermine, shikimate, *N*-acetylornithine, and 2-deoxyribose 1-phosphate (2dR1P), were significantly decreased during the test in the YM group compared to those in the placebo group. Figure 3c shows a heatmap visualizing the unsupervised hierarchical clustering of changes in these 20 metabolites for all individuals in each group.

### 3.5. Relationship between Fecal Metabolites and Sleep

To explore the relationship between sleep quality and gut environment, forward stepwise linear regression analysis was applied using 20 differential metabolites as independent valuables. The respective sleep parameters, TIB or N3 latency time, were used as dependent variables. As Table 4 shows, the extension of TIB could be explained at 53.0% by elevated propionate levels in feces. In contrast, the shortened N3 latency could be explained at 59.3% by increased GABA and dTMP levels. Even GABA concentration alone could also explain 33.3% of the change in N3 latency.

### 3.6. Safety

Changes in blood pressure, complete body composition, blood cell content, lipid parameters, renal function, and liver function were assessed. The change in Ht (%) was significantly lower in the YM group than in the placebo group (*p* = 0.024). The change in BUN (%) was significantly higher in the YM group than in the placebo group (*p* = 0.044). Nevertheless, both items were usually within the normal range for the Japanese population (Ht: 36–50%; BUN:8–20 mg/dL).

No side effects or severe or moderate AEs were observed during the intervention. Seventeen mild AEs were recorded during the study period. In the YM group, eleven AEs were recorded, including elevated ALT (*n* = 1), elevated γ-GTP (*n* = 1), elevated DBP (*n* = 1), adverse reactions to the COVID-19 vaccine (*n* = 5), headache (*n* = 1), and scratchiness (*n* = 2). In the placebo group, 6 AEs were recorded, including reduced WBC (*n* = 1), diarrhea (*n* = 2), back pain (*n* = 1), and adverse reactions to the COVID-19 vaccine (*n* = 2). The study investigator determined that none of the AEs were related to the consumption of the current test tablets.

## 4. Discussion

This study aimed to assess whether supplementation with YM would confer health benefits to healthy adults experiencing discomfort in defecation. Recently, we reported that the regular intake of YM for eight weeks improved the bowel habits of healthy women aged 30–49 years [9]. This study was conducted with male and female participants in a broader age range (22–64 years) than in our previous study, and the duration of intake was four weeks, shorter than in the previous study. In this study, daily consumption of YM for four weeks did not significantly impact the BSS scores but increased the frequency and volume of bowel movements in healthy adults with a tendency for constipation. The ingredient YM was shown to be a beneficial prebiotic as it improves some aspects of bowel habits.

Constipation can occur in anyone as a minor discomfort that may result from altered gut motility. Chronic constipation is estimated to affect about 10–15% of the population and significantly impacts the quality of life [33]. Multiple factors are essential for normal gut motility, such as the immune and nervous system, bile acid metabolism and mucus secretion, and the gut microbiota and metabolites [34]. An imbalance or dysfunction in any one of these factors can lead to abnormal gut motility and, consequently, constipation symptoms. Lately, the availability of some probiotics and prebiotics has been suggested to provide some relief from symptoms of constipation, apart from pharmaceuticals such as laxatives and serotonin 5-HT4 receptor agonists [33,34]. Interestingly, Zhu et al. [35] reported that the microbiota of constipated patients presents a decreased relative abundance of *Bacteroidetes*. Additionally, Kim et al. [36] reported that the relative abundance of *Bacteroides* in constipated patients was lower than in control, and its relative abundance had increased after treatment of constipation. Based on these findings, *Bacteroides* may play an important role in balancing the intestinal microflora and improving constipation. YM is a highly complex polysaccharide that is not assimilated by most organisms. *B. thetaiotaomicron*, one of the dominant bacterial species in the human gut microbiota, expresses specific α-mannan-degrading enzymes. Therefore, unlike other gut microbes, it can utilize YM as a carbon source [13]. In the present study, the relative abundance of *B. thetaiotaomicron* in feces was increased in the YM group, which is consistent with our previous study [9]. Recently, mutant-monocolonized gnotobiotic mice showed that the selective bile salt hydrolase (BSH) of *B. thetaiotamicron* can alter host bile acid composition [37]. In addition, using a human colonic microflora model, the YM administration was shown to promote the production of propionate with increasing *B. thetaiotaomicron*, known as a propiogenic bacteria [8,38]. Consistent with the in vitro study, YM ingestion elevated fecal propionate levels in the present study. Secondary bile acids and SCFAs, including acetate, propionate, and butyrate, could trigger the secretion of gut hormones, such as peptide YY (PYY), glucagon-like peptide-1 (GLP-1), and 5-hydroxytryptamine (5-HT) from intestinal epithelial cells [39,40,41]. Subsequently, these gut hormones can affect gut sensation, secretion, and motility, primarily through stimulating specific receptors which are located on epithelial cells, enteric neurons, and smooth muscle cells [42,43]. Thus, it can be speculated that YM-degrading microorganisms produce propionate in the gut, and the subsequent increase in intestinal propionate levels enhances gut motility.

More importantly, we found that supplementation with YM as a prebiotic could alter sleep architecture and contribute to better sleep quality. Recent studies have focused on the interaction between gut microbiota and sleep disturbances, indicating that a healthy gut environment, including microbiota and metabolite composition, is increasingly important for host health [44]. Several studies have shown that certain probiotics can enhance sleep quality in humans [45], but there are limited human studies on the sleep-improving effects of prebiotics. To the best of our knowledge, no study has reported a beneficial effect of prebiotics on objective sleep quality in humans. However, Saleh-Ghadimi et al. [21] reported that the consumption of resistant dextrin ameliorated subjective sleep quality in obese women with type 2 diabetes. Here, we showed for the first time that daily intake of YM significantly shortened N3 latency and lengthened N3 duration compared to placebo using an EEG device. N3, in which growth hormone (GH) is released in abundance, serves as an indicator of deep and restorative sleep [46]. Furthermore, YM intake was shown to increase TIB compared to placebo. Adequate sleep has been proposed to be important for daily functioning and long-term health. Shorter sleep has been reported to lead to heart disease, obesity, cognitive decline, a worse mood, and even a shorter life expectancy [47]. Our present results suggest that YM intake may improve some aspects of sleep quality, although the mechanism underlying the effect of YM on altering sleep architecture remains unclear.

We hypothesized that changes in the levels of certain metabolites in the colon following changes in the gut microbiota composition due to YM intake would be responsible for the effects of YM on sleep quality. We also evaluated the impact of YM intake on fecal metabolites using a CE-TOFMS-based metabolomics approach. As a result, 20 differential metabolites between the YM and placebo groups were screened. This study represents the first report on the alteration of fecal metabolites following YM administration. To explore the metabolites associated with changes in objective sleep parameters—TIB or N3 latency, which were significantly altered by YM ingestion—stepwise linear regression was performed using these twenty differential metabolites as independent variables. Relationships between the changes in fecal metabolites and each sleep variable were found. The extension of TIB could be explained in 53.0% by elevated propionate levels in feces. However, the shortened N3 latency could be explained at 59.3% by increased GABA and dTMP levels. Even GABA concentration alone could also explain 33.3% of the change in N3 latency. Based on the above, the effect of YM on sleep might be explained via at least two routes: gut-derived propionate and GABA.

First, regarding the association between sleep and fecal propionate level, a prior study in preschool-aged children found that the fecal propionate concentration was lower in children with long wake times after sleep onset, indicative of difficulty maintaining sleep [48]. SCFAs, including acetate, propionate, and butyrate, have been shown to induce intestinal production of 5-hydroxytryptophan (5-HTP) and serotonin (5-hydroxytryptamine, 5-HT) by stimulating tryptophan hydroxylase (TpH1) expression in the enterochromaffin cells [49]. 5-HTP, a precursor of 5-HT, crosses the blood–brain barrier, unlike 5-HT; therefore, gut-derived 5-HTP could contribute to the synthesis of 5-HT in the brain [50]. 5-HTP and its metabolites, 5-HT and melatonin, have been reported to influence gut motility, mood, and sleep/arousal [51]. Based on the above, it is hypothesized that stimulation of propionate contributes to a possible beneficial effect of YM on sleep quality.

In addition, we consider the possible effects of gut-derived GABA on sleep quality. Our results found that fecal GABA levels were increased by YM administration. GABA is a key inhibitory neurotransmitter closely related to sleep, and GABA receptors are pivotal targets for pharmaceuticals, such as benzodiazepine, to alleviate insomnia [52]. GABA-enriched food ingredients have also been shown to enhance sleep quality in patients with insomnia symptoms [53] and healthy individuals [54]. GABA has been reported to be produced by intestinal micro-organisms, such as *Lactobacillus*, *Bifidobacterium*, and *Bacteroides* [55,56]. In particular, *Bacteroides* were found to produce GABA in the pH range of the human colon. Hence, Bacteroides were suggested to be the main GABA-producing bacteria in the human large intestine [56]. The proton-coupled amino acid transporters-mediated GABA transport through the basolateral membrane on human intestinal Caco-2 cell monolayers was demonstrated by Nielsen et al. [57]. Therefore, gut-derived GABA is suggested to be capable of interacting with GABA receptors on afferent vagal nerves and signaling the central GABAergic system [44,57]. For some GABA-producing enteric bacteria, including *B. thetaiotamicron*, GABA secretion has been reported to function as an acid-resistance mechanism. GABA is exported from the cell in a protonated form, alkalizing the cytoplasm [58]. The increase in GABA in feces may be due to a defensive response to mitigate acidification by propionate produced by YM-degrading bacteria. Oral intake of YM may affect sleep quality via gut-derived propionic acid and GABA.

One limitation of this research is that the EEG data collection was partly incomplete. Incomplete EEG data due to inappropriate use of equipment reduced the sample size of the EEG data set, although this reduction is in line with the missing data observed in previous EEG studies. Although bowel habits may be affected by diet, we did not impose strict dietary restrictions on participants or provide uniform diets.

## 5. Conclusions

The present study was conducted to assess the effects of YM supplementation on bowel movements, fecal properties, and sleep quality, resulting in the following two key findings. First, this study among healthy adults with a tendency toward constipation revealed that daily consumption of YM did not bring about any modifications in their stool forms; however, it did result in an increase in both the frequency and volume of bowel movements. Second, we found that daily intake of YM had no significant effect on sleep efficiency or sleep latency, but it accelerated the transition to N3, meaning the deep sleep stage, and further lengthened the TIB and N3 duration. Our findings imply that YM may be a useful prebiotic for improving some aspects of gut health and sleep quality. Further studies are required to fully assess the beneficial effects of YM supplementation and clarify the underlying mechanism.

## Figures and Tables

**Table 1:** Participant’s background characteristics (per protocol set).

Parameters	Placebo	YM
Age, years	52.2 ± 7.3	48.1 ± 10.2
Gender, *n* (%)		
Male	4 (21.1)	6 (33.3)
Female	15 (78.9)	12 (66.7)
Alcohol drinking habits, *n* (%)		
No	9 (47.4)	8 (44.4)
Yes	10 (52.6)	10 (55.6)
Smoking habit, *n* (%)		
Non-smoker	12 (63.2)	12 (66.7)
Ex-smoker	4 (21.1)	3 (16.7)
(no smoking for > 6 months)		
Smoker	3 (15.8)	3 (16.7)
Fiber intake (g/day)	11.2 ± 3.3	12.5 ± 3.4

Numerical data are presented as mean ± standard deviation (SD). Categorical data are presented as numbers (percentages).

**Table 2:** Sleep measurements.

Parameters (Unit)	Group	Pre-Treatment		Post-Treatment		Changes	
		Values	*p*-Values	Values	*p*-Values	Values	*p*-Values
TIB (min)	Placebo	375.9 ± 13.9	0.355	375.5 ± 13.8	0.293	−0.5 ± 8.5	0.003 **
	YM	349.2 ± 18.9		404.3 ± 14.6		55.1 ± 18.1	
SE (%)	Placebo	75.8 ± 5.3	0.521	83.7 ± 2.1	0.239	7.9 ± 4.3	0.203
	YM	77.7 ± 3.8		76.8 ± 3.5		−0.9 ± 3.5	
N3 duration (min)	Placebo	44.0 ± 8.4	0.864	30.7 ± 5.5	0.022 *	−13.4 ± 9.9	0.152
	YM	48.1 ± 8.4		54.8 ± 9.4		6.7 ± 8.1	
SOL (min)	Placebo	30.0 ± 8.0	0.913	17.9 ± 3.8	0.501	−12.1 ± 9.0	0.239
	YM	23.1 ± 4.3		23.8 ± 5.9		0.8 ± 4.7	
N3 latency (min)	Placebo	55.1 ± 9.8	0.696	98.3 ± 27.3	0.008 **	43.2 ± 25.7	0.017 *
	YM	65.1 ± 16.8		37.1 ± 11.1		−28.1 ± 12.0	

Values are presented as mean ± SE. The *p*-values were measured using the Mann–Whitney U-test (* *p* < 0.05, ** *p* < 0.01). One participant in the placebo group, for whom EEG data were not available at baseline, was excluded from the analysis of sleep parameters. Abbreviations: TIB, total time in bed; SE, sleep efficiency; N3, non-REM sleep stage 3; SOL, sleep onset latency.

**Table 3:** Quantitative PCR.

Parameters (Unit)	Placebo			YM			*p*-Values ^1^
	Pre- Treatment	Post- Treatment	Changes	Pre- Treatment	Post- Treatment	Changes	
Total bacteria (10^9^ copies/g feces)	155.4 ± 17.3	170.9 ± 20.5	15.5 ± 20.4	182.1 ± 57.9	172.3 ± 26.5	−9.8 ± 52.4	0.649
*B. thetaiotaomicron* (10^9^ copies/g feces)	0.49 ± 0.23	0.31 ± 0.15	−0.18 ± 0.11	0.15 ± 0.05	0.31 ± 0.13	0.16 ± 0.12	0.046 *
Relative abundance of	0.31 ± 0.12	0.17 ± 0.07	−0.14 ± 0.08	0.09 ± 0.03	0.15 ± 0.05	0.07 ± 0.04	0.035 *
*B. thetaiotaomicron* (%)							

Values are presented as mean ± SE. ^1^ *p*-values indicate a comparison of changes from pre-treatment between the groups using the Student’s *t*-test (* *p* < 0.05).

**Table 4:** Forward stepwise regression analysis for TIB and N3 latency.

Dependent Variables	Steps	Independent Variables	R^2^	Adjusted R^2^	B ± SE	β	*p*-Values
TIB	1	Propionate	0.557	0.530	0.04 ± 0.01	0.747	<0.0001
N3 latency	1	GABA	0.372	0.333	−0.41 ± 0.13	−0.610	0.007
	2	GABA	0.641	0.593	−0.46 ± 0.11	−0.683	0.001
		dTMP			−4.60 ± 1.37	−0.524	0.004

Data are shown at TIB and N3 latency and include the independent valuables for each model. Abbreviations: TIB, total time in bed; N3, non-REM sleep stage 3.

## Supplementary Materials

The following supporting information can be downloaded at: https://www.mdpi.com/article/10.3390/nu16010141/s1, Table S1: Participant inclusion and exclusion criteria; Table S2: qPCR amplification conditions; Table S3: Bowel habits; Figure S1: PCA plot of all samples; Table S4: Significant differential metabolites between the groups.

## References

[1] Pacheco A.R., Moel M., Segrè D. (2019). Costless metabolic secretions as drivers of interspecies interactions in microbial ecosystems. Nat. Commun..

[2] Morowitz M.J., Carlisle E.M., Alverdy J.C. (2011). Contributions of Intestinal Bacteria to Nutrition and Metabolism in the Critically Ill. Surg. Clin. N. Am..

[3] D’Amelio P., Sassi F. (2018). Gut Microbiota, Immune System, and Bone. Calcif. Tissue Int..

[4] Agus A., Planchais J., Sokol H. (2018). Gut microbiota regulation of tryptophan metabolism in health and disease. Cell Host Microbe.

[5] Halverson T., Alagiakrishnan K. (2020). Gut microbes in neurocognitive and mental health disorders. Ann. Med..

[6] Farias D.P., de Araújo F.F., Neri-Numa I.A., Pastore G.M. (2019). Prebiotics: Trends in food, health and technological applications. Trends Food Sci. Technol..

[7] Oba S., Washida K., Shimada Y., Sunagawa T., Tanihiro R., Sugiyama H., Nakamura Y. (2020). Yeast mannan increases *Bacteroides thetaiotaomicron* abundance and suppresses putrefactive compound production in in vitro fecal microbiota fermentation. Biosci. Biotechnol. Biochem..

[8] Oba S., Sunagawa T., Tanihiro R., Awashima K., Sugiyama H., Odani T., Nakamura Y., Kondo A., Sasaki D., Sasaki K. (2020). Prebiotic effects of yeast mannan, which selectively promotes *Bacteroides thetaiotaomicron* and *Bacteroides ovatus* in a human colonic microbiota model. Sci. Rep..

[9] Tanihiro R., Sakano K., Oba S., Nakamura C., Ohki K., Hirota T., Sugiyama H., Ebihara S., Nakamura Y. (2020). Effects of yeast mannan which promotes beneficial *Bacteroides* on the intestinal environment and skin condition: A randomized, double-blind, placebo-controlled study. Nutrients.

[10] Liu H.Z., Liu L., Hui H., Wang Q. (2015). Structural characterization and antineoplastic activity of *Saccharomyces cerevisiae* mannoprotein. Int. J. Food Prop..

[11] Kocourek J., Ballou C.E. (1969). Method for fingerprinting yeast cell wall mannans. J. Bacteriol..

[12] Scheller H.V., Ulvskov P. (2010). Hemicelluloses. Annu. Rev. Plant Biol..

[13] Cuskin F., Lowe E.C., Temple M.J., Zhu Y., Cameron E., Pudlo N.A., Porter N.T., Urs K., Thompson A.J., Cartmell A. (2015). Human gut *Bacteroidetes* can utilize yeast mannan through a selfish mechanism. Nature.

[14] Hansen R., Sanderson I.R., Muhammed R., Allen S., Tzivinikos C., Henderson P., Gervais L., Jeffery I.B., Mullins D.P., O’Herlihy E.A. (2020). A double-blind, placebo-controlled trial to assess safety and tolerability of (Thetanix) *Bacteroides thetaiotaomicron* in adolescent Crohn’s disease. Clin. Transl. Gastroenterol..

[15] Billings M.E., Hale L., Johnson D.A. (2020). Physical and social environment relationship with sleep health and disorders. Chest.

[16] Orr W.C., Chen C.L. (2005). Sleep and the gastrointestinal tract. Neurol. Clin..

[17] Ko C.Y., Liu Q.Q., Su H.Z., Zhang H.P., Fan J.M., Yang J.H., Hu A.K., Liu Y.Q., Chou D., Zeng Y.M. (2019). Gut microbiota in obstructive sleep apneahypopnea syndrome: Disease-related dysbiosis and metabolic comorbidities. Clin. Sci..

[18] Lin A., Shih C.T., Huang C.L., Wu C.C., Lin C.T., Tsai Y.C. (2019). Hypnotic effects of *Lactobacillus fermentum* PS150TM on pentobarbital-induced sleep in mice. Nutrients.

[19] Ogawa Y., Miyoshi C., Obana N., Yajima K., Hotta-Hirashima N., Ikkyu A., Kanno S., Soga T., Fukuda S., Yanagisawa M. (2020). Gut microbiota depletion by chronic antibiotic treatment alters the sleep/wake architecture and sleep EEG power spectra in mice. Sci. Rep..

[20] Faul F., Erdfelder E., Lang A.G., Buchner A. (2007). G*Power 3: A flexible statistical power analysis program for the social, behavioral, and biomedical sciences. Behav. Res. Methods.

[21] Saleh-Ghadimi S., Dehghan P., Sarmadi B., Maleki P. (2022). Improvement of sleep by resistant dextrin prebiotic in type 2 diabetic women coincides with attenuation of metabolic endotoxemia: Involvement of gut brain axis. J. Sci. Food Agric..

[22] O’Donnell L.J., Virjee J., Heaton K.W. (1990). Detection of pseudodiarrhoea by simple clinical assessment of intestinal transit rate. BMJ.

[23] Ohashi Y., Hirayama A., Ishikawa T., Nakamura S., Shimizu K., Ueno Y., Tomita M., Soga T. (2008). Depiction of metabolome changes in histidine-starved *Escherichia coli* by CE-TOFMS. Mol. BioSyst..

[24] Ooga T., Sato H., Nagashima A., Sasaki K., Tomita M., Soga T., Ohashi Y. (2011). Metabolomic anatomy of an animal model revealing homeostatic imbalances in dyslipidaemia. Mol. BioSyst..

[25] Sugimoto M., Wong D.T., Hirayama A., Soga T., Tomita M. (2010). Capillary electrophoresis mass spectrometry-based saliva metabolomics identified oral, breast and pancreatic cancer-specific profiles. Metabolomics.

[26] MetaboAnalyst—Statistical, Functional and Integrative Analysis of Metabolomics Data. https://www.metaboanalyst.ca/.

[27] Hatanaka M., Yamamoto K., Suzuki N., Iio S., Takara T., Morita H., Takimoto T., Nakamura T. (2018). Effect of *Bacillus subtilis* C-3102 on loose stools in healthy volunteers. Benef. Microbes.

[28] Tong J., Liu C., Summanen P., Xu H., Finegold S. (2011). Application of quantitative real-time PCR for rapid identification of *Bacteroides fragilis* group and related organisms in human wound samples. Anaerobe.

[29] Furet J., Firmesse O., Gourmelon M., Bridonneau C., Tap J., Mondot S., Doré J., Corthier G. (2009). Comparative assessment of human and farm animal faecal microbiota using real-time quantitative PCR. FEMS Microbiol. Ecol..

[30] Araki T., Uemura T., Yoshimoto S., Takemoto A., Noda Y., Izumi S., Sekitani T. (2020). Wireless monitoring using a stretchable and transparent sensor sheet containing metal nanowires. Adv. Mater..

[31] Berry R.B., Budhiraja R., Gottlieb D.J., Gozal D., Iber C., Kapur V.K., Marcus C.L., Mehra R., Parthasarathy S., Quan S.F. (2012). Rules for scoring respiratory events in sleep: Update of the 2007 AASM Manual for the Scoring of Sleep and Associated Events. Deliberations of the Sleep Apnea Definitions Task Force of the American Academy of Sleep Medicine. J. Clin. Sleep Med..

[32] Matsumori S., Teramoto K., Iyori H., Soda T., Yoshimoto S., Mizutani H. (2022). HARU Sleep: A deep learning-based sleep scoring system with wearable sheet-type frontal EEG sensors. IEEE Access.

[33] Aziz I., Whitehead W.E., Palsson O.S., Törnblom H., Simrén M. (2020). An approach to the diagnosis and management of Rome IV functional disorders of chronic constipation. Expert Rev. Gastroenterol. Hepatol..

[34] Dimidi E., Christodoulides S., Scott S.M., Whelan K. (2017). Mechanisms of action of probiotics and the gastrointestinal microbiota on gut motility and constipation. Adv. Nutr..

[35] Zhu L., Liu W., Alkhouri R., Baker R.D., Bard J.E., Quigley E.M., Baker S.S. (2014). Structural changes in the gut microbiome of constipated patients. Physiol. Genom..

[36] Kim S.-E., Choi S.C., Park K.S., Park M.I., Shin J.E., Lee T.H., Jung K.W., Koo H.S., Myung S.-J., Constipation Research Group of Korean Society of Neurogastroenterology and Motility (2015). Change of fecal flora and effectiveness of the short-term VSL#3 probiotic treatment in patients with functional constipation. J. Neurogastroenterol. Motil..

[37] Yao L., Seaton S.C., Ndousse-Fetter S., Adhikari A.A., DiBenedetto N., Mina A.I., Banks A.S., Bry L., Devlin A.S. (2018). A selective gut bacterial bile salt hydrolase alters host metabolism. eLife.

[38] Porter N.T., Larsbrink J. (2022). Investigation and alteration of organic acid synthesis pathways in the mammalian gut symbiont *Bacteroides thetaiotaomicron*. Microbiol. Spectr..

[39] Appleby R.N., Walters J.R. (2014). The role of bile acids in functional GI disorders. Neurogastroenterol. Motil..

[40] Martin C.R., Osadchiy V., Kalani A., Mayer E.A. (2018). The Brain-Gut-Microbiome Axis. Cell. Mol. Gastroenterol. Hepatol..

[41] Wang J.K., Yao S.K. (2021). Roles of gut microbiota and metabolites in pathogenesis of functional constipation. Evid.-Based Complement. Altern. Med..

[42] Ge X., Zhao W., Ding C., Tian H., Xu L., Wang H., Ni L., Jiang J., Gong J., Zhu W. (2017). Potential role of fecal microbiota from patients with slow transit constipation in the regulation of gastrointestinal motility. Sci. Rep..

[43] Guarino M., Cheng L., Cicala M., Ripetti V., Biancani P., Behar J. (2011). Progesterone receptors and serotonin levels in colon epithelial cells from females with slow transit constipation. Neurogastroenterol. Motil..

[44] Wang Z., Wang Z., Lu T., Chen W., Yan W., Yuan K., Shi L., Liu X., Zhou X., Shi J. (2022). The microbiota-gut-brain axis in sleep disorders. Sleep Med. Rev..

[45] Yong S.J., Tong T., Chew J., Lim W.L. (2019). Antidepressive mechanisms of probiotics and their therapeutic potential. Front. Neurosci..

[46] Nishida K., Sawada D., Kuwano Y., Tanaka H., Rokutan K. (2019). Health benefits of Lactobacillus gasseri CP2305 tablets in young adults exposed to chronic stress: A randomized, double-blind, placebo-controlled study. Nutrients.

[47] Grandner M.A., Hale L., Moore M., Patel N.P. (2010). Mortality associated with short sleep duration: The evidence, the possible mechanisms, and the future. Sleep Med. Rev..

[48] Wang Y., van de Wouw M., Drogos L., Vaghef-Mehrabani E., Reimer R.A., Tomfohr-Madsen L., Giesbrecht G.F. (2022). Sleep and the gut microbiota in preschool-aged children. Sleep.

[49] Reigstad C.S., Salmonson C.E., Rainey J.F., Szurszewski J.H., Linden D.R., Sonnenburg J.L., Farrugia G., Kashyap P.C. (2015). Gut microbes promote colonic serotonin production through an effect of short-chain fatty acids on enterochromaffin cells. FASEB J..

[50] Margret A.A., Mareeswari R., Arun Kumar K., Jerley A.A. (2021). Relative profiling of L-tryptophan derivatives from selected edible mushrooms as psychoactive nutraceuticals to inhibit P-glycoprotein: A paradigm to contest blood-brain barrier. BioTechnologia.

[51] Heitkemper M.M., Han C.J., Jarrett M.E., Gu H., Djukovic D., Shulman R.J., Raftery D., Henderson W.A., Cain K.C. (2016). Serum tryptophan metabolite levels during sleep in patients with and without irritable bowel syndrome (IBS). Biol. Res. Nurs..

[52] Gottesmann C. (2020). GABA mechanisms and sleep. Neuroscience.

[53] Byun J.I., Shin Y.Y., Chung S.E., Shin W.C. (2018). Safety and efficacy of gamma-aminobutyric acid from fermented rice germ in patients with insomnia symptoms: A randomized, double-blind trial. J. Clin. Neurol..

[54] Yamatsu A., Yamashita Y., Maru I., Yang J., Tatsuzaki J., Kim M. (2015). The improvement of sleep by oral intake of GABA and Apocynum venetum leaf extract. J. Nutr. Sci. Vitaminol..

[55] Barrett E., Ross R.P., O’Toole P.W., Fitzgerald G.F., Stanton C. (2012). γ-Aminobutyric acid production by culturable bacteria from the human intestine. J. Appl. Microbiol..

[56] Strandwitz P., Kim K.H., Terekhova D., Liu J.K., Sharma A., Levering J., McDonald D., Dietrich D., Ramadhar T.R., Lekbua A. (2019). GABA-modulating bacteria of the human gut microbiota. Nat. Microbiol..

[57] Nielsen C.U., Carstensen M., Brodin B. (2012). Carrier-mediated γ-aminobutyric acid transport across the basolateral membrane of human intestinal Caco-2 cell monolayers. Eur. J. Pharm. Biopharm..

[58] Ikeyama N., Murakami T., Toyoda A., Mori H., Iino T., Ohkuma M., Sakamoto M. (2020). Microbial interaction between the succinate-utilizing bacterium Phascolarctobacterium faecium and the gut commensal *Bacteroides thetaiotaomicron*. MicrobiologyOpen.

