# Supplementary material for: Effects of Prebiotic Yeast Mannan on Gut Health and Sleep Quality in Healthy Adults: A Randomized, Double-Blind, Placebo-Controlled Study
Source: Nutrients. 2023 Dec 31;16(1):141. doi: 10.3390/nu16010141 (PMC10780920; doi:10.3390/nu16010141)
Supplement: Supplementary file 1 [file nutrients-16-00141-s001.zip › nutrients-2804373-supplementary.pdf]

Supplementary Information

Title

Effects of Prebiotic Yeast Mannan on Gut Health and Sleep Quality in Healthy Adults: A Randomized, Double-Blind, Placebo-Controlled Study

Reiko Tanihiro [1,*], Masahiro Yuki [1], Masaki Sasai [1], Akane Haseda[2], Hiroyo Kagami-Katsuyama[2], Tatsuhiko Hirota [1], Naoyuki Honma [2], and Jun Nishihira [2]

1 Core Technology Laboratories, Asahi Quality and Innovations, Ltd., Moriya 302-0106, Japan; masahiro.yuki@asahi-qi.co.jp (M.Y.); masaki.sasai@asahi-qi.co.jp (M.S.); tatsuhiko.hirota@asahi-qi.co.jp (T.H.)

2 Department of Medical Management and Informatics, Hokkaido Information University, Ebetsu 069-8585, Japan; nishihira@do-johodai.ac.jp

*Correspondence: reiko.tanihiro@asahi-qi.co.jp; Tel.: +81-297-46-9347

Table S1. Participant inclusion and exclusion criteria.

**Inclusion criteria**	
1	Participants who agreed to participate in this study with written informed consent
2	Participants with discomfort in defecation
**Exclusion criteria**	
1	Participants with previous or current history of gastrointestinal cancer
2	Participants with gastrointestinal disorders
3	Participants who use gastrointestinal drugs or female hormone once or more a week
4	Participants suffering from serious cerebrovascular, cardiac, renal, hepatic diseases or infectious diseases requiring reporting to the authorities
5	Participants with a history of major surgery to the digestive system
6	Participants with significant abnormalities in blood pressure measurements
7	Participants with severe anemia
8	Menopausal women with significant changes in physical condition
9	Participants who repeat constipation and diarrhea
10	Participants with defecation frequency of 3 or less/ 9 or more per week
11	Participants with stool characteristics (Bristol Stool Scale, BSS) of 5 or more
12	Participants who have a habit of consuming yogurt 6 days or more per week
13	Participants who have a habit of consuming beverages that include lactic acid bacteria and/or bifidobacteria 2 days or more per week
14	participants who have a habit of consuming beverages that include dietary fiber reinforced food and/or oligo sugar 1 day or more per week
15	Participants allergic to drugs or foods, especially yeast
16	Participants who used antibiotics within 12 weeks prior to this study
17	Alcohol abusers, heavy smokers, or participants with extremely irregular lifestyles
18	Participants who donated either 400 mL of whole blood within 12 weeks (men), 16 weeks (women), 200 mL of whole blood within 4 weeks, or blood components within 2 weeks before this study
19	Pregnant or possibly pregnant or lactating women
20	Participants scheduled to participate in another clinical trial between 4 weeks prior to the screening test and the end of intake
21	Any other participants deemed ineligible by the study investigator

Table S2. qPCR amplification conditions.

Target species	Primer-F<br>Primer-R (5' to 3')<br>Probe (5' FAM - 3' TAMRA)	Strains for standard curves	PCR amplification condition	Ref.
All eubacteria	CGGTGAATACGTTCCCGG<br>TACGGCTACCTTGTTACGACTT	*B. fragilis*<br>ATCC 25285	95 °C for 10 s,<br>[95 °C for 20 s, 56 °C for 20 s, and 72 °C for 30 s] × 40 cycles	29
B. thetaiotaomicron	GCAAACTGGAGATGGCGA<br>AAGGTTTGGTGAGCCGTTA<br>TCGATGGGGATGCGTTCCATTAGG	*B. thetaiotaomicron*<br>ATCC 29741	95 °C for 30 s,<br>[95 °C for 15 s and 62.5 °C for 60 s] × 40 cycles	28

Table S3. Bowel habits.

Parameters (Unit)	Placebo			YM			*p*-values[1]
	Pre-treatment	Post-treatment	Changes	Pre-treatment	Post-treatment	Changes	
Defecation frequency (times/day)	0.81 ± 0.05	0.83 ± 0.05	0.02 ± 0.05	0.75 ± 0.06	0.96 ± 0.06	0.21 ± 0.06	0.037*
Defecation volume (units/day)	5.27 ± 0.27	4.86 ± 0.33	−0.41 ± 0.33	4.86 ± 0.40	5.74 ± 0.30	0.88 ± 0.30	0.013*
Bristol stool scale	3.61 ± 0.10	4.01 ± 0.11	0.40 ± 0.11	3.45 ± 0.15	3.57 ± 0.11	0.12 ± 0.11	0.096

Values are presented as the means ± SE. [1]*p*-values indicate a comparison of changes from pre-treatment between groups by Student's t-test (*$p < 0.05$).

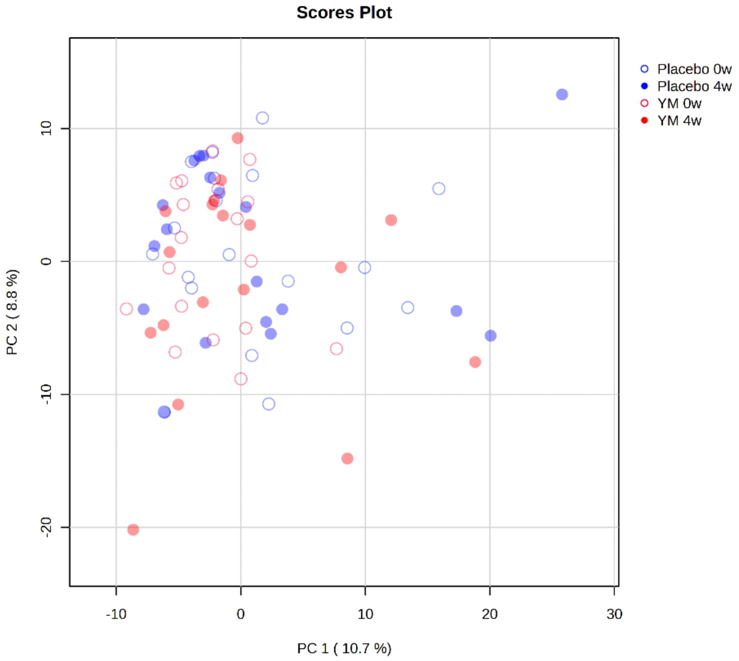

Figure S1. PCA plot of all samples. The YM group is depicted in red, and the placebo group in blue. Open circles represent the pre-treatment condition for each group (YM 0w, placebo 0w), and closed circles represent the post-treatment condition for each group (YM 4w, placebo 4w).

Table S4. Significant differential metabolites between the groups.

Metabolites	HMDB ID	PubChem CID	VIP	*p*-values	Changes
2-deoxyribose 1-phosphate (2dR1P)	HMDB0001351	5460448	1.91	0.048	↓
Castanospermine	HMDB0249700	54445	2.10	0.028	↓
N-Acetylornithine	HMDB0003357	439232	1.95	0.043	↓
Cyprodinil	HMDB0034853	86367	2.26	0.018	↓
Methylguanidine	HMDB0001522	10111	2.25	0.018	↓
Prostaglandin E_2	HMDB0001220	5280360	2.44	0.010	↓
Shikimate	HMDB0003070	8742	2.07	0.031	↓
Deoxyadenosine monophosphate (dAMP)	HMDB0000905	12599	2.29	0.016	↑
Deoxycytidine monophosphate (dCMP)	HMDB0001202	13945	2.20	0.021	↑
Deoxythymidine monophosphate (dTMP)	HMDB0001227	9700	1.98	0.040	↑
Trimethylamine	HMDB0000906	1146	1.97	0.041	↑
Gamma-aminobutyric acid (GABA)	HMDB0000112	119	2.12	0.027	↑
Taurine	HMDB0000251	1123	1.96	0.041	↑
Propionate	HMDB0000237	1032	2.13	0.026	↑
N^1-Acetylspermidine	HMDB0001276	496	2.42	0.011	↑
S-Adenosylmethionine (SAM)	HMDB0001185	34755	2.31	0.015	↑
Creatinine	HMDB0000562	588	2.40	0.011	↑
Mannosamine		440049	2.04	0.033	↑
Cystine	HMDB0000192	595	2.50	0.008	↑
Glycyl-glycine (Gly-Gly)	HMDB0011733	11163	2.31	0.015	↑

HMDB, Human Metabolome Database; VIP, VIP values for the first component of PLS-DA. The *p*-values were calculated using Student's t-test. An upward arrow (↑) indicates an increase in the YM group compared to the placebo group. A downward arrow (↓) indicates a decrease in the YM group compared to the placebo group.
